# Effects of short-term metyrapone treatment on glycaemic control in patients with mild autonomous cortisol secretion: a pilot study

**DOI:** 10.1007/s12020-026-04601-y

**Published:** 2026-05-02

**Authors:** Alessandro Rossini, Silvia Pellegrini, Silvia Ippolito, Sara Cassibba, Luca Giovanelli, Margherita Zappoli, Roberto Trevisan, Giuseppe Lepore

**Affiliations:** 1 Department of Medicine and Surgery, University of Milano Bicocca, Milan, Italy; 2 Endocrinology and Diabetes Unit, Papa Giovanni XXIII Hospital, Bergamo, Italy

**Keywords:** MACS, Hypercortisolism, Mild Autonomous Cortisol Secretion, Metyrapone, Continuous Glucose Monitoring

## Abstract

**Purpose:**

Mild autonomous cortisol secretion (MACS) is associated with impaired glucose metabolism, but the metabolic effects of adrenostatic therapy in this setting remain unclear. This study aimed to explore whether changes in CGM-derived glycaemic metrics could be observed during timed evening metyrapone administration in patients with MACS.

**Methods:**

This prospective, single-centre interventional study included nine patients with MACS due to adrenal adenoma or hyperplasia and coexisting prediabetes or untreated mild type 2 diabetes. Participants underwent continuous glucose monitoring (CGM) for one week at baseline and for one week during treatment with the 11β-hydroxylase inhibitor metyrapone (500 mg at 6:00 PM and 250 mg at 10:00 PM). Morning (8:00 AM) serum cortisol, ACTH levels and parameters of glucose metabolism were assessed. Data distribution was evaluated using the Shapiro–Wilk test; paired comparisons were performed using appropriate parametric or non-parametric tests, with correction for multiple comparisons.

**Results:**

After one week of metyrapone treatment, the percentage of time with glucose levels between 70 and 140 mg/dL (time-in-tight-range, TITR) increased from 81% to 84% (p < 0.05). Time in range (70–180 mg/dL) increased from 98.4% to 99.4%, while time above range (> 180 mg/dL) decreased from 1.4% to 0.1% (both p < 0.05). ACTH concentrations increased, while morning cortisol levels remained stable.

**Conclusions:**

After short-term evening administration of metyrapone a modest yet statistically significant change in CGM-derived glycaemic metrics was observed in patients with MACS, without altering morning cortisol levels. These findings support the need for further studies to evaluate the long-term metabolic effects of adrenostatic therapy in this population.

Mild autonomous cortisol secretion (MACS) is a condition characterised by adrenocorticotropic hormone (ACTH)-independent cortisol hypersecretion, without the signs and symptoms of overt Cushing’s syndrome, associated with an adrenal adenoma or hyperplasia [1].

MACS is associated with various metabolic and cardiovascular comorbidities, ultimately contributing to an increased risk of mortality. A significant association has been observed between MACS and glucose metabolism abnormalities, due to cortisol’s effect in promoting insulin resistance and decreasing pancreatic insulin secretion. As a result, the prevalence of type 2 diabetes mellitus (T2DM) is increased in patients with MACS [2].

Adrenalectomy is effective in improving the metabolic profile in patients with unilateral MACS [2, 3] and is currently considered a treatment option for selected patients with MACS and relevant comorbidities [1]. Medical therapy may be appropriate for patients with bilateral forms or unsuitable for surgery [4]. Evidence regarding the medical therapy of MACS remains limited. In two case series [5, 6], mifepristone, an oral, non-selective glucocorticoid receptor antagonist, has shown efficacy in improving insulin sensitivity in patients with MACS. However, its use is limited by adverse effects and its application in the management of MACS is currently not recommended [7]. More recently, Oda et al. [8] evaluated the effect of an 11β-hydroxysteroid dehydrogenase type 1 inhibitor in a mixed population of patients with Cushing syndrome (CS) and MACS, reporting an improvement in the area under curve (AUC) of plasma glucose after 12 weeks, but not after 24 weeks of treatment.

There is clear evidence that metyrapone, a steroidogenesis inhibitor, can improve glucose metabolism in patients with CS [9, 10]. However, to the best of our knowledge, no studies have investigated the potential metabolic benefits of metyrapone in patients with MACS. Interestingly, a recent study by Debono et al. [11] demonstrated that the use of timed evening doses of metyrapone restored the circadian rhythm of cortisol secretion in patients with MACS, resulting in a reduction in the cardiovascular risk marker IL-6. We designed the present study to explore short-term CGM-derived glycaemic changes during evening-timed metyrapone administration in patients with MACS. Continuous glucose monitoring (CGM) was employed alongside standard serum glycaemic parameters, as CGM is regarded as the gold standard for assessing glucose variability and overall glycaemic control. Unlike blood glucose or glycated haemoglobin measurements obtained from venous or capillary samples, which reflect single time points or long-term averages, CGM provides a continuous assessment of glucose levels, capturing real-time fluctations, peaks, and nadirs throughout the day and night.

## Materials and methods

Patients with unilateral or bilateral adrenal incidentalomas with benign imaging features, referred to the Endocrinology Department of Papa Giovanni XXIII Hospital in Bergamo between December 2023 and February 2025, were screened for MACS using the 1-mg overnight dexamethasone suppression test (DST). A diagnosis of MACS was established based on a post-DST serum cortisol concentration > 1.8 µg/dL in two separate test, in accordance with current ESE guidelines [1].

During the screening visit, detailed information was collected regarding personal history of cortisol-related comorbidities (arterial hypertension, cardiovascular disease, diabetes mellitus, dyslipidemia, osteoporosis/osteopenia, and nephrolithiasis). Anthropometric measurements (including blood pressure, height, and weight) were recorded. In addition, a resting electrocardiogram (ECG) was performed, and fasting blood samples were collected for the determination of plasma glucose, HbA1c, insulin, ACTH, cortisol, and potassium levels. Serum cortisol concentrations were measured using a chemiluminescent immunoassay (Siemens Healthineers); the lower limit of quantification (LoQ) of the assay was *≤* 0.31 µg/dL, with a measuring interval of 0.5–75 µg/dL, according to the manufacturer’s specifications.

Eligible patients included those with a post-DST cortisol > 1.8 µg/dL, confirmed on two separate test. ACTH-independency was evaluated by measuring basal morning ACTH levels, and was defined as basal morning ACTH < 10 pg/mL. In addition, in one patient with indeterminate ACTH levels (14.7 pg/mL), ACTH measurement was repeated after 1 mg DST. Post-DST ACTH was below the assay’s lower limit of quantification (< 3 pg/mL), and it was considered consistent with ACTH-independency. Participants were also required to have impaired glycaemic control, defined as a fasting plasma glucose ≥ 100 mg/dL or an HbA1c ≥ 39 mmol/mol, including newly diagnosed and treatment naïve type 2 diabetes, prediabetes, or impaired fasting glucose. Exclusion criteria included overt hypercortisolism, uncontrolled hypertension (systolic blood pressure ≥ 140 mmHg and/or diastolic blood pressure ≥ 90 mmHg, despite pharmacological treatment), uncontrolled diabetes, QT interval abnormalities, hypokalemia, or pregnancy.

Informed consent was obtained from all participants. Nine eligible patients were consecutively enrolled in the study. Each participant underwent two periods of CGM, each lasting one week, using the FreeStyle Libre 2 sensor (Abbott Diabetes Care, CA). CGM was firstly assessed the week before the initiation of metyrapone therapy. The second CGM assessment commenced on the day following the start of metyrapone treatment, administered at a total daily dose of 750 mg (500 mg at 6:00 PM and 250 mg at 10:00 PM). Participants were instructed to maintain their usual lifestyle, including dietary habits and physical activity, throughout the study duration. No structured lifestyle, dietary, or sleep interventions were implemented during the study.

After 7 days of metyrapone treatment, participants underwent a repeat clinical evaluation, including blood pressure measurement, assessment of blood tests (glucose, insulin, cortisol, ACTH, potassium), evaluation of treatment tolerability, and sensor removal. CGM data from both the pre-treatment and treatment periods were downloaded via the LibreView platform. The following CGM metrics were calculated: Time in Range (TIR, 70–180 mg/dL), Time in Tight Range (TITR, 70–140 mg/dL), Time Above Range (TAR, > 180 mg/dL), Time Below Range (TBR, < 70 mg/dL), mean sensor glucose, and the coefficient of variation (CV) of mean sensor glucose.

Statistical analyses included descriptive statistics (mean ± SD or medians with interquartile ranges (IQR) for continuous variables; frequencies and percentages for categorical variables). Normality of data distribution was formally tested using the Shapiro–Wilk test. Comparisons between baseline and post-treatment values were performed using paired Student’s t-test for normally distributed variables and the Wilcoxon signed-rank test for non-normally distributed variables. Because multiple paired tests were performed, p-values were adjusted for multiple comparisons using the Benjamini–Hochberg (BH) procedure to control the false discovery rate (FDR). Both unadjusted and BH-adjusted p-values are reported. A two-tailed adjusted p value < 0.05 was considered statistically significant.

To quantify the magnitude of the observed changes beyond statistical significance, effect sizes were computed as Cohen’s d for paired differences, calculated as the mean difference divided by the standard deviation of the difference. Values of 0.2, 0.5, and 0.8 were interpreted as small, moderate, and large effects, respectively.

## Results

A total of 14 patients were initially screened for eligibility. Three patients with known type 2 diabetes receiving glucose-lowering therapy were excluded. Among the remaining subjects, three were not enrolled because they were considered unable to reliably comply with study procedures or declined participation. Consequently, nine patients (5 females, age 64.4 ± 10.3 years) were included in the study. Clinical characteristics are detailed in Table 1. Mean body mass index (BMI) was 31.4 ± 5.8 kg/m^2^. Five patients (55.6%) had a unilateral adenoma, 3 (33.3%) had bilateral hyperplasia, and 1 (11.1%) had unilateral hyperplasia. Seven patients were treated with antihypertensive agents, and five received lipid-lowering therapy. At baseline one participant was found to have undiagnosed type 2 diabetes, and 8 met the diagnostic criteria for prediabetes (HbA1c 39–47 mmol/mol, or fasting plasma glucose 100–125 mg/dL). Baseline median HbA1c was 44.7 ± 3.5 mmol/mol.

**Table 1:** Baseline clinical, anthropometric and biochemical features of patients. Units of measurement are reported for each variable within the table. *BMI*: body mass index; *DST*: dexamethasone suppression test; *ACTH*: adrenocorticotropic hormone

Patient	Gender	Age (years)	Weight (kg)	Height (cm)	BMI (kg/m^2^)	Adrenal pathology	Cortisol after DST (µg/dL)	ACTH (pg/ml)
1	M	54	107	173.0	35.8	Unilateral adenoma	3.3	3.3
2	M	56	130	182.0	39.3	Unilateral adenoma	2.5	10.0
3	M	73	76	165.5	27.7	Bilateral hyperplasia	3.4	14.7
4	M	79	85	170.5	29.2	Unilateral hyperplasia	2.6	9.8
5	F	78	64	159.0	25.3	Unilateral adenoma	2.3	9.9
6	F	69	82	144.0	39.6	Bilateral hyperplasia	4.2	9.4
7	F	57	73	170.0	25.3	Unilateral adenoma	2.5	9.1
8	F	55	90	164.5	33.3	Bilateral hyperplasia	3.6	8.8
9	F	59	71	162.0	27.0	Unilateral adenoma	3.6	7.4

After one week of treatment with metyrapone, mean fasting plasma glucose decreased (111.6 vs. 105.4 mg/dL, *p* < 0.05). Median serum ACTH levels increased (9.4 vs. 16.3 pg/mL, *p* < 0.05) while serum cortisol (25.7 vs. 25.4 µg/dL) and mean potassium levels remained stable (4.6 vs. 4.4 mEq/L) (Table 2).

**Table 2:** Biochemical parameters of patients before and after one week of metyrapone treatment. Units of measurement are reported for each variable within the table. Data are expressed as mean ± SD for normally distributed variables and as median (IQR) for non-normally distributed variables, based on Shapiro–Wilk testing. Comparisons between baseline and post-treatment values were performed using paired Student’s t-test for normally distributed variables and the Wilcoxon signed-rank test for non-normally distributed variables. Correction for multiple testing was performed using the Benjamini–Hochberg procedure. *HbA1c*: glycated hemoglobin; *HOMA-IR*: homeostatic model assessment of insulin resistance; *ACTH*: adrenocorticotrophic hormone

	Baseline	Post-treatment	*p*-value
Glycaemia (mg/dL)	111.6 ± 16.5	105.4 ± 11.9	0.13
HbA1c (mmol/mol)	44.0		
Insulinemia (µIU/mL)	13.2 (8.7–18.55)		
HOMA-IR	3.9 (2.1–5.05)		
Cortisol (µg/dL)	25.7 ± 11.38	25.4 ± 8.53	0.79
ACTH (pg/mL)	9.4 (8.1–9.95)	16.3 (11.2–24.25)	0.046*
Potassium (mEq/L)	4.6 (4.22–4.78)	4.4 ± 0.37	0.09

After correction for multiple testing with the Benjamini–Hochberg procedure, ACTH remained statistically significant (BH-adjusted *p* ≈ 0.046), whereas the change in glucose did not remain statistically significant (BH-adjusted *p* ≈ 0.13). Effect sizes ranged from negligible to large across parameters. Insulin and HOMA-IR showed small effects (Cohen’s d ≈ − 0.3 and − 0.4), cortisol showed a negligible effect (d ≈ − 0.1), and potassium showed a moderate effect (d ≈ − 0.7). Glucose and ACTH exhibited large effect sizes (d ≈ − 0.8 and + 1.1, respectively).

**Fig. 1:**
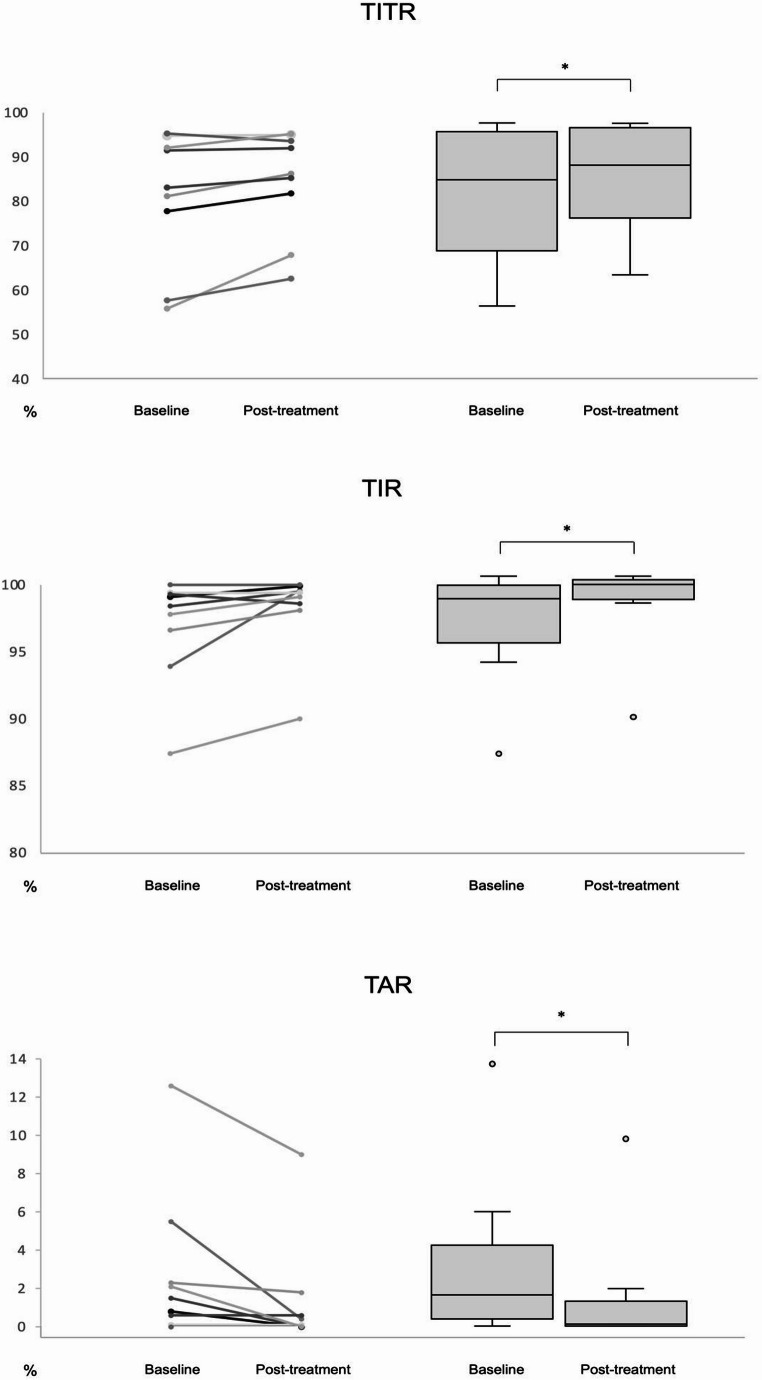
Continuous glucose monitoring (CGM) metrics at baseline and after one week of metyrapone treatment. Dots indicate individual participants. Lines connecting paired baseline and post-treatment values highlight within-subject trajectories. Box plots represent the median and interquartile range. Paired comparisons between baseline and post-treatment values were performed using the Wilcoxon signed-rank test. * *p* < 0.05. TITR: Time in Tight Range (70–140 mg/dL); TIR: Time in Range (70–180 mg/dL); TAR: Time Above Range (> 180 mg/dL)

Continuous glucose monitoring (CGM) metrics are presented in Fig. 1. At baseline, median Time in Range (TIR; 70–180 mg/dL) was 98.4%, median Time in Tight Range (TITR; 70–140 mg/dL) was 83.1%, median Time Above Range level 1 (TAR 1; >180 mg/dL) was 1.4%, and mean CV was 9.55%. After one week of metyrapone therapy, both TITR and TIR showed a statistically significant increase, reaching 86.2% and 99.4%, respectively (both *p* < 0.05). TAR 1 significantly decreased to 0.1% (*p* < 0.05), and CV slightly decreased to 9.32%. No episodes of Time Above Range level 2 (TAR 2; glucose > 250 mg/dL) were recorded at any time point. Mean sensor glucose exhibited a modest decline, from 121.4 mg/dL at baseline to 120.1 mg/dL following treatment.

After correction for multiple testing with the Benjamini–Hochberg procedure, the observed differences remained statistically significant (adjusted p values: 0.03 for all tests). Effect sizes ranged from moderate to large across parameters. TITR displayed a moderate effect (d ≈ 0.55), whereas TIR and TAR showed moderate-large effects (d ≈ 0.74 and 0.87, respectively).

Blood pressure values remained stable during the study period, with no clinically relevant changes observed at follow-up. Treatment was generally well tolerated. One participant reported mild asthenia, nausea and dizziness during the first days of treatment, which resolved spontaneously without treatment discontinuation. No serious adverse events were recorded.

## Discussion

The optimal management of patients with MACS remains a matter of debate, particularly for those who are not suitable candidates for surgery. In this setting, our study investigated the effects of metyrapone, usually employed in the treatment of more severe forms of hypercortisolism, on glucose metabolism in this specific patient population.

MACS is characterised by a disruption of the circadian rhythm of cortisol secretion, resulting in increased evening and nocturnal cortisol exposure. In a 2017 study, Debono et al. [11] reported that patients with MACS exhibit significantly higher evening and nighttime serum cortisol levels compared to both patients with non-functioning adrenal incidentalomas and healthy controls. More recently, a metabolomic analysis by Saini [12] et al. showed that individuals with MACS have reduced urinary steroid day/night ratios and elevated evening total and free cortisol levels relative to controls. This inappropriated evening cortisol exposure has been linked to impaired glucose tolerance and reduced insulin sensitivity [13], as well as to an increase in other cardiovascular risk factors [14]. Debono et al. demonstrated that metyrapone – a cortisol synthesis inhibitor with rapid onset of action – effectively reduces evening and nocturnal cortisol levels when administered at scheduled evening doses, without affecting morning cortisol concentrations [11]. In the same study, normalisation of the circadian cortisol rhythm was associated with a reduction in circulating levels of interleukin-6, an established cardiovascular risk factor [11].

The findings of the present study are consistent with the hypothesis that metyrapone may influence cortisol rhythmicity in patients with MACS. It should be noted that a single morning serum cortisol measurement may fail to capture clinically relevant alterations in cortisol circadian rhythm, particularly in conditions such as MACS, where cortisol excess predominantly affects the evening and nocturnal periods. Accordingly, the absence of significant changes in morning serum cortisol observed in our study is consistent with the deliberate choice of evening-timed metyrapone administration, aimed at reducing inappropriate nocturnal cortisol exposure while preserving morning cortisol secretion. This approach reflects a chronotherapeutic strategy targeting cortisol rhythmicity rather than absolute cortisol suppression and has previously demonstrated advantages in the management of hypercortisolism, including in patients with Cushing’s disease [15]. In line with this interpretation, the observed increase in ACTH levels in the presence of stable morning cortisol levels is compatible with a redistribution of cortisol secretion across the 24-hour cycle. Future studies incorporating 24-hour urinary free cortisol, late-night salivary cortisol, or more frequent serum cortisol sampling will be required to confirm this mechanism directly.

In our patients, metyrapone treatment was associated with modest but statistically significant changes across all CGM-derived metrics of glycaemic control, including Time in Tight Range (TITR), Time in Range (TIR), and Time Above Range (TAR). Among these, the increase in TITR deserves particular attention, as this parameter is currently considered the most sensitive indicator of glucose regulation in healthy, non-diabetic individuals [16]. At baseline, TITR values were already relatively high, indicating only mild impairment of glucose metabolism; after one week of metyrapone treatment, TITR increased further, accompanied by a parallel increase in TIR and a reduction in TAR. Although statistically significant, these changes were small in absolute terms, and their clinical relevance remains uncertain, particularly in a population with minimal dysglycaemia at baseline. Indeed, healthy individuals typically exhibit TITR values close to 96% [16], and the improvements observed in our cohort do not approach this physiological range nor suggest a meaningful restoration of glucose homeostasis. Rather than reflecting a clinically relevant metabolic correction, these findings are more likely to represent an early and transient effect of reduced evening and nocturnal cortisol exposure on glucose excursions.

This interpretation is consistent with the short duration of treatment and with the concept that CGM is sensitive to subtle and rapid changes in glucose dynamics, even before sustained metabolic adaptations occur. The concurrent improvement in TIR and TAR, despite already optimal baseline values, further supports the high sensitivity of CGM metrics to modest shifts in cortisol exposure. Importantly, the observation of coherent changes across multiple CGM parameters over such a short timeframe reinforces the hypothesis that cortisol chronomodulation can influence glucose variability; however, whether these early effects translate into clinically meaningful metabolic benefits remains to be determined. In this setting, it is worth highlighting that a three-month treatment with mifepristone, a glucocorticoid receptor antagonist, was associated with a decrease in fasting glucose and insulin resistance in patients with MACS [5]. Similarly, a 24-week treatment with an 11β-HSD1 inhibitor resulted in improved insulin sensitivity and favourable changes in body composition in a heterogeneous cohort of patients with MACS or Cushing’s syndrome [8].

These data support the rationale for future, adequately powered studies evaluating the longer-term metabolic impact of metyrapone.

In our cohort, metyrapone was generally well tolerated; only one patient reported mild asthenia, nausea, and dizziness, which did not require discontinuation of therapy. These symptoms occurred despite normal serum cortisol concentrations and were likely attributable to glucocorticoid withdrawal syndrome, a condition that may arise following cessation of exposure to supraphysiological levels of endogenous or exogenous glucocorticoids [17]. Metyrapone has also been associated with worsening of hypertension and hypokalaemia, both related to accumulation of glucocorticoid precursors with mineralocorticoid activity. In our cohort, potassium levels and blood pressure values remained substantially stable.

Several limitations of this study should be acknowledged. These include the small sample size, which limits statistical power and generalisability, and the short duration of treatment, which precluded assessment of longer-term metabolic outcomes such as HbA1c. In addition, lifestyle-related factors such as sleep and stress were not objectively assessed, and steroidogenesis precursors were not measured during metyrapone therapy. Nevertheless, the study also has notable strengths, including its prospective design, well-defined inclusion criteria, and the use of CGM, which provides detailed 24-hour glucose profiling. Overall, the present study was intentionally designed as an exploratory pilot investigation; therefore, the absence of a control group and the short treatment duration do not allow confirmatory conclusions regarding the metabolic effects of metyrapone but rather support hypothesis generation to be tested in larger, adequately powered controlled studies.

In conclusion, our findings indicate that short-term metyrapone therapy is associated with a modest improvement in CGM-derived indices of glycaemic control in patients with MACS. These findings should be interpreted cautiously and considered hypothesis-generating rather than confirmatory. Larger controlled studies with longer treatment durations are needed to establish whether the early metabolic changes observed after one week translate into clinically meaningful and sustained benefits. From a translational perspective, chronomodulated metyrapone therapy may represent a potential medical option in selected patients with MACS, either as a temporary strategy while awaiting adrenal surgery or as a potential longer-term approach in individuals who are not surgical candidates, including those with bilateral disease or significant comorbidities.

## Data Availability

The datasets used and/or analysed during the current study are available from the corresponding author on reasonable request.

## Abbreviations

AUC: Area under curve
BMI: Body mass index
CGM: Continuous glucose monitoring
CS: Cushing syndrome
CV: Coefficient of variation
DST: Dexamethasone suppression test
ECG: Electrocardiogram
HbA1c: Glycated Haemoglobin
HOMA-IR: Homeostatic Model Assessment of Insulin Resistance
MACS: Mild autonomous cortisol secretion
ACTH: Adrenocorticotropic hormone
T2DM: Type 2 diabetes mellitus
TAR: Time Above Range
TBR: Time Below Range
TIR: Time in Range
TITR: Time in Tight Range
